# A cross-sectional study of secondhand smoke exposure and respiratory symptoms in non-current smokers in the U.S. trucking industry: SHS exposure and respiratory symptoms

**DOI:** 10.1186/1471-2458-13-93

**Published:** 2013-02-01

**Authors:** Francine Laden, Yueh-Hsiu Chiu, Eric Garshick, S Katharine Hammond, Jaime E Hart

**Affiliations:** 1 Department of Environmental Health, Harvard School of Public Health, Boston, MA, USA; 2 Department of Epidemiology, Harvard School of Public Health, Boston, MA, USA; 3 Channing Division of Network Medicine, Department of Medicine, Brigham and Women’s Hospital and Harvard Medical School, Boston, MA, USA; 4 Pulmonary and Critical Care Medicine Section, Medical Service, VA Boston Healthcare System, Boston, MA, USA; 5 Environmental Health Sciences Division, School of Public Health, University of California, Berkeley, CA, USA

**Keywords:** Occupational health, Secondhand smoke, Respiratory symptoms

## Abstract

**Background:**

Previous studies have suggested associations of adult exposures to secondhand smoke (SHS) with respiratory symptoms, but no study has focused on blue-collar industrial environments. We assessed the association between SHS and respiratory symptoms in 1,562 non-current smoking U.S. trucking industry workers.

**Methods:**

Information on SHS exposure and respiratory health was obtained by questionnaire. Multiple logistic regression analyses were used to assess the associations of recent and lifetime exposures to SHS with chronic phlegm, chronic cough, and any wheeze, defined by American Thoracic Society criteria.

**Results:**

In analyses adjusted for age, gender, race, childhood SHS exposure, former smoking, pack-years of smoking and years since quitting, body mass index, job title, region of the country, and urban residence, recent exposures to SHS were associated with all three respiratory symptoms (odds ratio (OR) = 1.46; 95% confidence interval (CI) = 1.00-2.13) for chronic cough, 1.55 (95% CI = 1.08-2.21) for chronic phlegm, and 1.76 (95% CI = 1.41-2.21) for any wheeze). Workplace exposure was the most important recent exposure. Childhood exposure to SHS was also associated with all three symptoms, but only statistically significantly for chronic phlegm (OR = 1.84; 95% CI = 1.24-2.75). Additional years of living with a smoker were associated with an increased risk, but there was no evidence of a dose–response, except for chronic phlegm.

**Conclusions:**

In this group of trucking industry workers, childhood and recent exposures to SHS were related to respiratory symptoms.

## Background

Secondhand smoke (SHS) is a complex mixture of chemicals generated by the burning of tobacco and the smoke exhaled by smokers [1]. Previous studies have suggested that adult exposure to SHS may be associated with adverse respiratory effects including acute and chronic respiratory symptoms, chronic rhinosinusitis, and asthma [1-10]; furthermore, the burden of respiratory symptoms on health related quality of life may be even stronger than that of lung function [11]. Epidemiologic studies of SHS exposure and respiratory symptoms have mainly considered office environments or service workers (in bars, restaurants, and casinos), but no study that we know of has focused on blue-collar industrial environments. Smoking prevalence in adults has been declining in the U.S. [12-14], but among the major blue-collar occupations it remains higher than that of many other occupational groups [15-20], suggesting that this is a population that still has high potential for SHS exposure [21]. Our previous work also found that despite state and local movements toward smoke-free laws, U.S. trucking industry workers were still exposed to workplace SHS as recently as 2005 [22]. In this study, we examined the association between SHS exposure and self-reported chronic respiratory symptoms, including cough, phlegm and wheeze, in a population of non-smoking employees in the U.S. unionized trucking industry.

## Methods

### Study subjects

In 2004 we mailed a health questionnaire to 3,689 members of the International Brotherhood of Teamsters, working or recently retired (within 7 years) from three large U.S. trucking companies. These individuals were all respondents to a previous questionnaire focused on active smoking habits [23]. We were unable to relocate 104 individuals and received responses from 2,294. The participants represented the major job categories in the trucking industry: long-haul driver (driving between cities), pick-up/delivery (P&D) driver (local driving within cities), dock worker (moving freight within the terminal), combination worker (workers who work both on the dock and drive P&D trucks), mechanic (repairing tractors and trailers), hostler (moving trucks in the terminal yard), and clerk (office worker). The study protocol was approved by the Brigham and Women’s Hospital, the Harvard School of Public Health and VA Boston Institutional Review Boards and consent was inferred from return of the completed questionnaires.

### Respiratory symptoms and illness

Prevalence of chronic respiratory symptoms was identified based on self-reports using questions from the American Thoracic Society DLD-78 questionnaire that includes questions on timing and severity of the symptoms [24]. “Chronic cough” was defined as cough on most days for 3 consecutive months or more during the year. “Chronic phlegm” was defined as phlegm on most days for 3 consecutive months or more during the year. “Any wheeze” was any report of wheezing with a cold or occasionally apart from colds or on most days or nights. Information on other respiratory illnesses, including hay fever, chronic bronchitis, emphysema, chronic rhinitis, chronic obstructive pulmonary disease (COPD), and asthma, was also obtained. In sensitivity analyses, we excluded all participants with existing doctor diagnosed chronic respiratory disease.

### Secondhand smoke exposure

We examined several metrics of SHS exposure. First, we used a previously validated question on exposure to SHS in the past 7 days from all sources. Workers were classified as “exposed” if they reported “yes” to “In the past 7 days, have you been exposed to someone else’s tobacco smoke?”. We also obtained information on recent home and recreational SHS exposures during an average week, as well as workplace exposure in the past 2 work days, and used this information to identify the likely sources of current SHS exposure. Recent home exposure was identified by the question “Has anyone (not including you) smoked tobacco in your home in the past 7 days?” (yes/no). Recreational and workplace exposures were identified based on time spent at places where people were smoking (restaurants, bars, clubs, other people’s homes, sports arena, concerts, etc.) in an average week, and amount of time spent in areas at work where smoking was allowed in the past 2 work days. Individuals were considered non-exposed if they did not spend time at smoking allowed areas or if they spent time there but reported no one was smoking.

We also examined two measures of chronic SHS exposure. Childhood SHS exposure was defined by whether the individual had ever lived with at least one smoker during their childhood (yes/no). Long-term exposure to SHS at home was identified by lifetime total years of residency with at least one smoker, and was categorized into four groups (<10, 10–19, 20–29, and ≥30 years).

### Statistical analysis

We used multiple logistic regression and estimated the odds ratio (OR) and 95% confidence intervals (95% CI) to evaluate the association between each SHS exposure metric and chronic cough, chronic phlegm, and any wheeze in separate models. We examined recent and long-term exposure to SHS in separate and combined models.

We considered, as potential confounders, variables that were either biologically meaningful or statistically significant in univariate analyses. These factors included childhood SHS exposure (in models of current exposure), previous smoking history (status, pack years smoked, and years since quit for former smokers), age, gender, race (white vs. nonwhite), job title, U.S. Census region (South, West, Midwest, Northeast), urban/non-urban residence, body mass index (BMI; weight divided by height squared, kg/m^2^), and self-reported medical history. Job title was included to control for differences in occupational exposures to air pollutants [25] and unmeasured lifestyle characteristics, and was categorized into four groups: drivers, terminal workers (dock workers and hostlers), office workers, and retired. Furthermore, we assessed effect modification on the relationship between current, childhood, and lifetime SHS exposure and respiratory symptoms by former smoking, childhood or current SHS exposure (as appropriate), race, obesity (BMI ≥30 kg/m^2^), and urban residence using stratified analyses and interaction terms. All analyses were performed in the SAS statistical package (version 9.1.3, SAS Institute Inc., Cary, NC).

## Results

### Participant characteristics

Only never smokers or former smokers who had quit smoking more than 10 years before completing the questionnaire and who completed the question on exposure to SHS in the past 7 days were eligible for analysis (n = 1,562). Participant characteristics, current and childhood SHS exposure, as well as prevalence of respiratory health outcomes are presented in Table 1. The study population was predominantly Caucasian, had a median age of 54 years and a median BMI of 29.0 kg/m^2^. About 63% of participants reported that they were exposed to SHS in the past 7 days and 70% were exposed to SHS during their childhood. There were 9.8% who reported chronic cough, 11.7% chronic phlegm, 47.8% any wheeze, and 50.8% with any symptoms.

**Table 1:** **Characteristics and SHS exposure of 1**,**562 non**-**current smoking workers in the U**.**S**. **unionized trucking industry**

**Continuous variables**	**Median (IQR)**	
Age (years)	54 (48–61)	
BMI (kg/m^2^)	29.0 (25.8-31.3)	
Total years lived with smokers (years)	20 (7–30)	
Pack-years of active smoking*	15 (5–30)	
Years since quit smoking*	22 (17–30)	
**Categorical Variables**	**N**	**(%)**
Male	1331	85.2
Caucasian	1404	89.9
Former smoker	692	44.3
Job title		
Terminal worker	203	13.0
Driver	791	50.6
Office worker	228	14.6
Retired	308	19.7
Region of Residency		
South	494	31.6
West	278	17.8
Midwest	521	33.4
Northeast	269	17.2
Living in urban area	1034	66.2
Exposed to SHS in the past 7 days	990	63.4
Current SHS exposure		
Recent home exposure	158	10.4
Recent workplace exposure	413	30.6
Recent recreational exposure	1127	80.5
Childhood SHS exposure	1077	70.1
Respiratory symptoms		
Chronic cough	153	9.8
Chronic phlegm	183	11.7
Any wheeze	718	47.8
Any symptom	793	50.8

IQR = interquartile range; BMI = body mass index.

* Among never smokers and former smokers who quit at least 10 years before completing the questionnaire.

### SHS exposure and respiratory symptoms

The results from the multivariable logistic regression models assessing the association of the different exposure metrics with each respiratory symptom are presented in Table 2. Adjusting for childhood SHS exposure and other potential confounders, exposure to SHS in the past 7 days was statistically significantly related to all symptoms. When simultaneously examining current SHS exposure by different locations/sources, workplace exposure and recreational exposures were the most important. Childhood SHS exposure was associated with an elevated risk of all symptoms, but was only statistically significantly associated with an increased risk of chronic phlegm. Lifetime SHS exposure at home was positively associated with all three symptoms.

**Table 2:** **Association between SHS exposure and respiratory symptoms among 1**,**562 non**-**smoking workers **^**a**^

**SHS exposure**	**Chronic cough**		**Chronic phlegm**		**Any wheeze**	
	**OR**	**95% CI**	**OR**	**95% CI**	**OR**	**95% CI**
*Total recent exposure*^b^						
Exposed to SHS in past 7 days	1.46	1.00 - 2.13	1.55	1.08 - 2.21	1.76	1.41 - 2.21
*Current exposure by location*^b,c^						
Recent workplace exposure^d^	1.74	1.14 - 2.67	1.75	1.16 - 2.65	1.20	0.90 - 1.60
Recent home exposure^e^	0.74	0.37 - 1.48	0.76	0.41 - 1.42	0.90	0.60 - 1.35
Recent recreational exposure^f^	0.91	0.55 - 1.50	1.24	0.74 - 2.09	2.03	1.45 - 2.83
*Childhood exposure*						
Lived with ≥1 smoker during childhood	1.21	0.81 - 1.80	1.84	1.24 - 2.75	1.16	0.91 - 1.47
*Life*-*time home exposure*						
Less than 10 years	Ref	--	Ref	--	Ref	--
10-19 years	1.39	0.84 - 2.31	1.86	1.15 - 3.03	1.21	0.90 - 1.63
20-29 years	1.75	1.04 - 2.94	1.92	1.16 - 3.18	1.17	0.85 - 1.60
at least 30 years	1.35	0.79 - 2.32	1.91	1.15 - 3.17	1.30	0.94 - 1.79
	*p* trend = 0.29		*p* trend = 0.02		*p* trend = 0.12	

^a^ Logistic regression models adjusted for age, sex, race, job title, former smoking, pack-years, years since quitting, BMI, region of country, and urban residence among never smokers or former smokers who quit at least 10 years before completing the questionnaire.

^b^ Additionally adjusted for childhood exposure to SHS.

^c^ Location specific exposures were all modeled simultaneously.

^d^ Exposure at work in the past 2 work days.

^e^ Recent exposure at home in an average week.

^f^ Recent exposure outside of home and work in an average week.

Note: trend analyses are estimated using the median value of each category.

### Effect modification

In stratified analyses (Table 3) we observed stronger associations of SHS exposure in the last 7 days with chronic cough and phlegm among those without childhood exposure to SHS and with chronic phlegm for those currently living in a non-urban area, although only the interaction term for urban/rural difference for chronic phlegm was statistically significant. On the other hand, we found stronger relationships of childhood SHS exposure with all respiratory symptoms among obese workers and those currently living in urban areas. The interactions were statistically significant between childhood SHS exposure and obesity for any wheeze (*p* = 0.02). In stratified analyses of the effects of lifetime home SHS exposure (Table 4), there was little evidence of effect modification, except by obesity, with stronger relationships among obese participants. In models restricted to individuals without chronic respiratory disease, results were similar (data not shown).

**Table 3:** **Effect modifications on the association between SHS exposure and respiratory symptoms among 1**,**562 non**-**current smoking workers **^**a**^

**Exposure metric**	**Stratification factors**	**Chronic cough**			**Chronic phlegm**			**Any wheeze**		
		**n**	**OR**	**95% CI**	**n**	**OR**	**95% CI**	**n**	**OR**	**95% CI**
Current Exposure	Former smoking									
	No	870	1.62	0.97 - 2.69	870	1.48	0.92 - 2.38	840	1.78	1.32 - 2.39
	Yes	692	1.31	0.73 - 2.33	692	1.69	0.98 - 2.93	662	1.73	1.21 - 2.45
	Obesity (BMI ≥30 kg/m^2^)									
	No	1033	1.68	1.04 - 2.73	1033	1.27	0.81 - 1.98	995	1.72	1.31 - 2.26
	Yes	522	1.19	0.64 - 2.22	522	2.06	1.11 - 3.83	500	1.99	1.33 - 2.99
	Currently in urban area									
	No	528	2.39	1.14 - 4.99	528	2.97	1.46 - 6.01	506	2.07	1.40 - 3.05
	Yes	1034	1.24	0.78 - 1.95	1034	1.19	0.78 - 1.83	996	1.67	1.26 - 2.22
	Childhood SHS exposure									
	No	459	2.31	1.06 - 5.02	459	2.42	1.06 - 5.53	437	1.41	0.94 - 2.13
	Yes	1077	1.24	0.80 - 1.93	1077	1.38	0.92 - 2.06	1040	1.97	1.50 - 2.60
Childhood Exposure	Former smoking									
	No	870	1.18	0.71 - 1.95	870	1.95	1.15 - 3.31	840	1.06	0.78 - 1.45
	Yes	692	1.16	0.60 - 2.23	692	1.57	0.85 - 2.92	662	1.24	0.84 - 1.83
	Obesity (BMI ≥30 kg/m^2^)									
	No	1033	0.86	0.54 - 1.39	1033	1.57	0.96 - 2.59	995	0.92	0.69 - 1.23
	Yes	522	2.63	1.21 - 5.75	522	2.63	1.31 - 5.29	500	1.93	1.25 - 2.95
	Currently in urban area									
	No	528	0.86	0.43 - 1.71	528	1.15	0.60 - 2.19	506	1.08	0.71 - 1.62
	Yes	1034	1.50	0.90 - 2.51	1034	2.47	1.43 - 4.25	996	1.24	0.92 - 1.68
	Current SHS exposure									
	No	572	1.80	0.83 - 3.93	572	2.71	1.18 - 6.20	550	0.89	0.60 - 1.33
	Yes	990	1.04	0.65 - 1.66	990	1.63	1.02 - 2.60	952	1.29	0.95 - 1.75

^a^ Logistic regression model adjusted for age, sex, race, job title, former smoking, pack-years, years since quitting, BMI, region of country, and urban residence except the stratification factor for each model, and also adjusted for either current exposure to SHS or childhood SHS exposure among never smokers or former smokers who quit at least 10 before completing the questionnaire.

* p for interaction < 0.05.

**Table 4:** **Effect modification of the association between lifetime home SHS exposure and respiratory symptoms **^**a**^

**Stratification factors**	**Life**-**time home exposure**	**Chronic cough**			**Chronic phlegm**			**Any wheeze**		
**Former smoking**		**OR**	**95% CI**	**p**	**OR**	**95% CI**	**p**	**OR**	**95% CI**	**p**
Yes	Less than 10 years	ref			ref			ref		
	10-19 years	2.25	0.83 - 6.12		1.96	0.83 - 4.65		1.16	0.68 - 1.97	
	20-29 years	2.47	0.94 - 6.48		2.09	0.91 - 4.78		1.18	0.71 - 1.96	
	at least 30 years	1.81	0.68 - 4.82		2.18	0.97 - 4.93		1.26	0.77 - 2.07	
No	Less than 10 years	ref			ref			ref		
	10-19 years	1.04	0.56 - 1.91		1.79	0.99 - 3.24		1.21	0.84 - 1.74	
	20-29 years	1.37	0.70 - 2.67		1.71	0.88 - 3.56		1.10	0.72 - 1.69	
	at least 30 years	1.17	0.58 - 2.35		1.54	0.77 - 3.10		1.33	0.86 - 1.69	
Obesity (BMI ≥30 kg/m^2^)										
Yes	Less than 10 years	ref			ref			ref		*
	10-19 years	3.42	1.18 - 9.90		2.45	1.07 - 5.59		1.55	0.91 - 2.63	
	20-29 years	5.99	1.99 - 18.02		2.32	0.95 - 5.56		1.88	1.04 - 3.40	
	at least 30 years	4.78	1.61 - 14.23		2.84	1.21 - 6.70		2.27	1.28 - 4.03	
No	Less than 10 years	ref			ref			ref		
	10-19 years	1.03	0.56 - 1.89		1.61	0.87 - 2.99		1.11	0.77 - 1.59	
	20-29 years	1.12	0.60 - 2.10		1.78	0.96 - 3.33		1.00	0.68 - 1.46	
	at least 30 years	0.77	0.39 - 1.53		1.49	0.77 - 2.87		1.07	0.72 - 1.58	
Currently in urban area										
Yes	Less than 10 years	ref			ref			ref		
	10-19 years	1.65	0.87 - 3.11		2.25	1.19 - 4.22		1.47	1.01 - 2.15	
	20-29 years	1.81	0.94 - 3.48		2.31	1.21 - 4.42		1.19	0.80 - 1.78	
	at least 30 years	1.35	0.69 - 2.67		1.94	1.01 - 3.75		1.47	0.99 - 2.19	
No	Less than 10 years	ref			ref			ref		
	10-19 years	0.88	0.35 - 2.22		1.26	0.57 - 2.79		0.90	0.55 - 1.49	
	20-29 years	1.78	0.72 - 4.42		1.37	0.58 - 3.22		1.16	0.67 - 2.01	
	at least 30 years	1.47	0.58 - 3.73		1.99	0.85 - 4.62		1.13	0.64 - 1.98	
Current SHS exposure										
Yes	Less than 10 years	ref			ref			ref		
	10-19 years	1.39	0.77 - 2.52		1.75	0.99 - 3.09		1.43	0.97 - 2.11	
	20-29 years	1.13	0.60 - 2.14		1.41	0.77 - 2.58		1.39	0.92 - 2.10	
	at least 30 years	1.02	0.55 - 1.89		1.32	0.74 - 2.37		1.19	0.81 - 1.76	
No	Less than 10 years	ref			ref			ref		
	10-19 years	1.53	0.52 - 4.48		2.86	1.00 - 8.16		1.01	0.63 - 1.64	
	20-29 years	5.21	1.89 - 14.42		3.94	1.37 - 11.33		0.88	0.51 - 1.50	
	at least 30 years	2.84	0.87 - 9.24		5.38	1.69 - 17.15		1.31	0.71 - 2.40	

^a^ Logistic regression model adjusted for age, sex, race, job title, former smoking, pack-years, years since quitting, BMI, region of country, and urban residence except the stratification factor for each model among never smokers or former smokers who quit at least 10 years before completing the questionnaire.

* p for interaction < 0.05.

## Discussion

In this population of non-smoking blue-collar trucking industry workers, we observed statistically significant relationships of recent SHS exposure with self-reported respiratory symptoms, including chronic cough, chronic phlegm, and any wheeze. Workplace appeared to be an important source of current exposure to SHS that influenced the workers’ respiratory health. In addition, childhood exposure to SHS was statistically significantly associated with chronic phlegm, whereas life-time duration of SHS exposure at home was positively associated with chronic phlegm and any wheeze. We also found evidence of effect modification by obesity and urban residence on the association between childhood SHS exposure and respiratory symptoms.

Our findings are fairly consistent with results from previous studies, although the evidence has been less conclusive in studies of adults than in studies of children. Studies have considered many combinations of SHS exposure experienced in childhood, adulthood, at home, at work, and outside of home or work. In addition, multiple outcome measures have been assessed (e.g. cough, phlegm, wheeze, chest tightness, breathlessness/dyspnea, headache, sinusitis, etc.), either by self-reports or doctor diagnosis. Therefore, it is difficult to make a concise comparison of studies.

Studies evaluating relationships between home SHS exposures (i.e. living with a smoker as an adult) and respiratory symptoms have observed inconsistent results, ranging from no association to an almost four-fold risk of chronic or usual cough [4,9,26-31]. In our study, no associations between home exposure and symptoms were found after controlling for current workplace exposure, but there was a significant association between total duration of life-time home exposure and chronic phlegm. Fewer studies have evaluated the effect of childhood SHS exposure on adult respiratory symptoms [26,32-34]. In a study of never smoking Singaporeans aged 45–74 years, positive associations were observed for childhood exposure with chronic phlegm and cough in adulthood [26]. A multicenter study in India showed positive association between exposure during childhood and both during childhood and adulthood with the prevalence of asthma [34]. A study in Western Norway revealed a significantly positive association between maternal smoking in childhood and wheeze in adulthood, but the results were not statistically significant for phlegm and cough [32]. The association between childhood exposure and adult wheeze was also observed in a multi-area European study, but this association was no longer statistically significant when restricted to never smokers [33]. In our present study of non-current smoking workers, childhood SHS exposure had a significant association with chronic phlegm and a borderline association with wheeze, after adjusting for current SHS exposure.

Analyses of self-reported workplace exposures have revealed more consistent associations with elevated risks of respiratory symptoms [6,9,35,36]. In a prospective diary study as part of the SAPALDIA study in never smoking Swiss adults, SHS exposure at work was associated with bronchitis symptoms (cough or phlegm) and asthmatic symptoms (wheeze, dyspnea, or chest tightness) [35]. Another study in 559 casino workers in London observed a significant relationship of intensity of workplace SHS exposure with shortness of breath and wheeze, controlling for home exposure [36]. Among workers in Spanish hospitality venues (bars, restaurants, hotels, and discotheques), respiratory symptoms decreased significantly [−98.1% (95% CI: -51% to −99.9%) in multivariate analysis] after smoking was totally banned in their workplace, whereas there was no significant change among workers employed at hospitality venues where only partial smoking bans or no restrictions were implemented [37]. A study of Norwegian restaurant employees found a complete smoking ban in restaurants contributed to fewer reports of respiratory symptoms [38]. A small cross-sectional study in the former Yugoslav Republic of Macedonia found a significantly higher prevalence of wheezing and breathlessness in nonsmoking women who worked in offices with smokers as opposed to those who did not [39]. A study of Italian women found exposure at work resulted in higher adjusted odds of wheeze, shortness of breath and asthma symptoms [9]. A prospective telephone survey in Hong Kong focusing on workplace SHS exposure reported elevated risks of frequent cough and phlegm for those exposed at work compared to those unexposed [6]. Our study also found workplace SHS exposure to have the strongest effect on respiratory symptoms, as compared to home exposure or recreational exposure.

Workers in the trucking industry are exposed to occupational sources of airborne particles and gasses. Therefore, there is the potential for confounding by or synergism with other exposures on the association of SHS exposure with health outcomes. Previous evidence has shown that SHS exposure is independently associated with respiratory symptoms and decreased lung function after adjusting for other sources of indoor particulate pollutants (e.g. wood stoves, candles, and gas cooking) in the general Danish population [5] and Singaporean women [40]. In this study we observed significant associations between SHS exposure and respiratory symptoms even with adjustment for job title, a fairly reasonable indicator of occupational exposure to air pollutants in this industry [25].

We recognize that this study has several limitations. It is a cross-sectional survey, limiting our ability to assess causality. The response rate, although comparable to other occupational studies [6,36], was only 64% of the people who received the second questionnaire. However, the responders and nonresponders were similar in terms of age, race, job title and active smoking. Both the exposures and the outcomes were self reported; therefore, the potential that recall bias could be responsible for our positive results cannot be ignored. In a study of workers in the same trucking companies we previously have shown evidence that self-reported workplace exposures to SHS are predictive of vapor-phase nicotine exposure as measured by a passive monitor worn on the lapel [22], providing evidence of validity of the self report of workplace exposure. It is unlikely that exposure only in the last 7 days would be causally related to chronic respiratory symptoms. However, it is likely that the recent exposure is reflective of more chronic exposures in the workplace, home and recreational facilities. Finally, there might be confounding by socioeconomic status and/or social stress. However, our study population is a group of blue-collar workers with fairly homogeneous backgrounds, and additional adjustment for educational level did not influence the results (data not shown). Thus, social factors are not likely to affect the results observed in this study.

## Conclusions

In this population of non-smoking blue-collar workers, self-reported SHS exposure is associated with self-reported chronic cough, chronic phlegm, and any wheeze. The strongest associations were observed with exposures experienced in the workplace. After adjusting for current SHS exposure, childhood exposure is associated with chronic phlegm. This adds to the body of literature supporting the importance of ensuring smoke-free workplaces.

## Abbreviations

SHS: Secondhand smoke; BMI: Body mass index.

## Competing interests

The authors declare that they have no competing interests.

## Authors’ contributions

FL conceived of the study, designed the statistical analysis, interpreted the results and drafted the manuscript. Y-HC performed the statistical analysis and helped to draft the manuscript. EG and SKH participated in the design of the study and the interpretation of the results. JEH participated in the design and coordination of the study, the statistical analysis, interpretation of results, and drafting of the manuscript. All authors read and approved the final manuscript.

## Pre-publication history

The pre-publication history for this paper can be accessed here:

http://www.biomedcentral.com/1471-2458/13/93/prepub
